# Disease control across urban–rural gradients

**DOI:** 10.1098/rsif.2020.0775

**Published:** 2020-12-09

**Authors:** Konstans Wells, Miguel Lurgi, Brendan Collins, Biagio Lucini, Rowland R. Kao, Alun L. Lloyd, Simon D. W. Frost, Mike B. Gravenor

**Affiliations:** 1 Department of Biosciences, Swansea University, Swansea SA2 8PP, UK; 2 Department of Mathematics, Swansea University, Swansea SA2 8PP, UK; 3 Swansea University Medical School, Swansea University, Swansea SA2 8PP, UK; 4 Department of Public Health and Policy, University of Liverpool, Liverpool L69 3GB, UK; 5 Health and Social Services Group, Welsh Government, Cardiff CF10 3NQ, UK; 6 Royal (Dick) Veterinary School of Veterinary Studies, University of Edinburgh, Edinburgh EH25 9RG, UK; 7 Biomathematics Graduate Program and Department of Mathematics, North Carolina State University, Raleigh, NC 27695, USA; 8 Microsoft Research Lab, Redmond, Washington, WA 98052, USA; 9 London School of Hygiene and Tropical Medicine, London WC1E 7HT, UK

**Keywords:** disease spread, epidemiological metapopulation dynamics, pandemic control, source–sink dynamics

## Abstract

Controlling the regional re-emergence of severe acute respiratory syndrome coronavirus 2 (SARS-CoV-2) after its initial spread in ever-changing personal contact networks and disease landscapes is a challenging task. In a landscape context, contact opportunities within and between populations are changing rapidly as lockdown measures are relaxed and a number of social activities re-activated. Using an individual-based metapopulation model, we explored the efficacy of different control strategies across an urban–rural gradient in Wales, UK. Our model shows that isolation of symptomatic cases or regional lockdowns in response to local outbreaks have limited efficacy unless the overall transmission rate is kept persistently low. Additional isolation of non-symptomatic infected individuals, who may be detected by effective test-and-trace strategies, is pivotal to reducing the overall epidemic size over a wider range of transmission scenarios. We define an ‘urban–rural gradient in epidemic size' as a correlation between regional epidemic size and connectivity within the region, with more highly connected urban populations experiencing relatively larger outbreaks. For interventions focused on regional lockdowns, the strength of such gradients in epidemic size increased with higher travel frequencies, indicating a reduced efficacy of the control measure in the urban regions under these conditions. When both non-symptomatic and symptomatic individuals are isolated or regional lockdown strategies are enforced, we further found the strongest urban–rural epidemic gradients at high transmission rates. This effect was reversed for strategies targeted at symptomatic individuals only. Our results emphasize the importance of test-and-trace strategies and maintaining low transmission rates for efficiently controlling SARS-CoV-2 spread, both at landscape scale and in urban areas.

## 1. Introduction

In the absence of a vaccine against coronavirus disease 2019 (COVID-19) during the initial pandemic phase, stakeholders are confronted with challenging decision-making to balance the constraints of social interaction and the efficient isolation of infectious individuals with economic and social pressures. There is now growing scientific evidence of how different containment strategies compare with each other amid the challenges of asymptomatic disease transmission and the ongoing need for improved estimates of epidemiological key parameters [1,2]. Non-pharmaceutical interventions for curbing the spread of severe acute respiratory syndrome coronavirus 2 (SARS-CoV-2) rely on the isolation of infectious individuals or general social distancing policies to reduce interactions between undetected infectious individuals and those susceptible to the disease. During uncontrolled pandemic spread, a central aim is to reduce case incidence in order to release the pressure on health systems. A more fundamental, long-term goal should be to reduce the overall epidemic size and allow particularly those most prone to suffer from the disease to escape infection until a pharmaceutical measure such as a vaccine is in place.

Control strategies are likely to be regional, and temporal, aiming to reduce the time-dependent reproduction number *R* while accepting that ongoing transmission is long term.

But how should these regional and temporary strategies account for disease spread in ever-changing transmission landscapes? One particular question faced by many countries is how do different control strategies differ in their efficacy in preventing disease spread across urban–rural gradients of different population densities and connectivity in urban and rural landscapes?

The spread of infectious disease is rarely random. It is instead likely to be driven by the complex and heterogeneous social interaction patterns of humans and the stark gradient between urban and rural populations. In a landscape context, contact opportunities within and among populations across urban–rural gradients and source–sink dynamics arising from infectious individuals encountering pools of susceptible individuals are the ultimate drivers of disease spread. Disease spread is thus hampered if contact opportunities are lower in poorly mixed populations [3–5]. Heterogeneity in contact patterns of individuals and among social groups is also assumed to impact the depletion of the pool of susceptible individuals and the build-up of possible herd immunity that prevent further spread [6,7]. Hence, future short- and long-term mitigation strategies that focus on managing regional and erratic outbreaks would benefit from a better understanding of which control strategies provide the best possible outcome under variable regional conditions.

**Figure 1.**
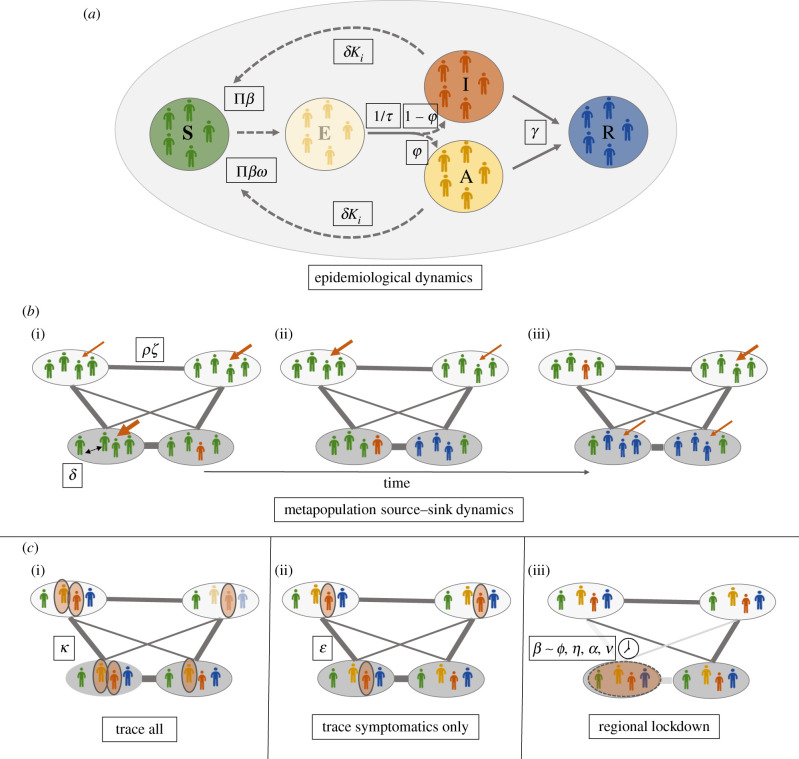
Key principles of epidemiological SARS-CoV-2 spread and control measures in a metapopulation context. In our framework, epidemiological dynamics are modelled using a stochastic individual-based model (*a*), in which susceptible individuals (S; green) are at risk of infection according to the number of contact symptomatic individuals (I; red), who may transmit the virus with transmission rate *β*, and asymptomatic individuals (A; dark yellow), who may transmit the virus with the scaled transmission rate *βω*. If infected, exposed individuals (E; light yellow) become infectious with or without symptoms (after the incubation period *τ*, individuals may either remain asymptomatic with probability φ or become symptomatic with probability 1 − φ); once recovered (R; blue), individuals no longer contribute to the virus transmission cycle. We conceptualized a landscape comprising a collection of rural and urban populations (upper and lower ovals in (*b*)) connected into a metapopulation network (*b*). Individuals may visit any other population as commuter travellers, whereby travel frequency is quantified by an overall commuter travel frequency (*ρ*), scaled by distance between populations (*ζ*) and motivated by the ‘gravity' of population distribution (in a gravity model individuals tend to travel to population clusters or those nearby (see text)). Within populations, contact frequencies might be density dependent (*δ*). Possible metapopulation source–sink dynamics (*b*) of disease spread in ever-changing disease landscapes may explain why metapopulation dynamics drive disease spread and also the efficacy of control measures in different populations (ovals in (*b*)). This occurs when, through time (from (*b*)(i) to (*b*)(iii)), populations that are strongly connected to other populations according to their proximity/gravity (as represented by the width of the connecting bars between local populations; dark-grey ovals represent urban, light-grey ovals rural populations) are at higher risk of future outbreaks given the spread of the virus and available pools of susceptible individuals. In (*b*), the width of the red arrows from above represents the susceptibility of populations to outbreaks based on their connectivity to other infected populations. During the course of the epidemic, those populations with previous outbreaks might be less prone to outbreaks because of reduced pools of susceptible individuals (e.g. (*b*)(ii)), while for a population without any previous outbreak the risk may constantly change according to the overall disease landscape and its dynamics (e.g. local population on the top right of the networks displayed in (*b*); compare the width of the red arrows). Colours of individuals within populations follow those of the S–E–A–I–R model in (*a*). In this study, we modelled the concept of test-and-trace control strategies, which involve the isolation of all infectious individuals (disease states, E, A, I) with efficacy *κ*, according to the proportion of infectious individuals successfully traced and isolated (*c*(i)). An alternative concept of control strategy is the isolation of symptomatic individuals (I) only with efficacy *ɛ* (*c*(ii)). Regional lockdown control strategies involve reducing the local transmission rate *β* in response to regional outbreaks of threshold level *α* according to a certain proportion of the regional population being in disease state I (*c*(iii)). Lockdown stringency *ϕ* determines how much the transmission rate is reduced. Lockdowns are of duration *η* and may include travel bans, which we modelled as a maximum distance *ν* from which individuals are allowed to visit a locked-down population. Red ovals around individuals/populations represent isolated individuals and locked-down populations, respectively. For all control scenarios, some infectious individuals may escape control measures, which can drive further disease spread within and among populations.

To the best of our knowledge, there is so far little evidence of how various disease control strategies differ in their efficacy across urban–rural gradients [8]. To address this gap, using an individual-based metapopulation model, we explore the outcomes of different control strategies to contain the epidemic size of COVID-19 in ever-changing disease landscapes of case numbers and susceptible depletion, which involve strong urban–rural gradients (see the illustration of the study concept in figure 1).

Our modelling approach is strategic, in contrast to many tactical COVID-19 simulation models that have focused on the replication of specific characteristics of real outbreaks with the aim of predicting the epidemic in specific locations [1,9,10]. Rather than focusing the modelling on a particular set of conditions, we aim to define a wide range of scenarios and explore the model behaviour across a large array of combinations of transmission and control parameters. In this way, we account for potential uncertainty in epidemiological parameters and degree of efficacy in ongoing and prospective control strategies. The influence of each parameter on particular outcomes can then be explored statistically. In this manner, we aim to highlight how the basic properties of realistic metapopulations' structures that include urban–rural gradients can affect the impact of control measures.

## 2. Methods

### 2.1. Case study of a rural–urban metapopulation in Wales

In order to provide an empirical basis to explore possible SARS-CoV-2 spread across an urban–rural gradient and the efficacy of different disease control measures, we selected four counties in southwestern Wales (Pembrokeshire, Carmarthenshire, Swansea, Neath Port Talbot) with a total human population size of 701 995 (hereafter termed the ‘metapopulation') dispersed over an area of 4811 km^2^ as a case study. This area was selected because of its strong urban–rural gradient, from city centres to sparsely occupied farming localities, and readily available demographic data. We used demographic data from the UK 2011 census (Office for National Statistics, 2011; www.ons.gov.uk), and constructed a metapopulation model at the level of a lower layer super-output area (LSOA), which provided *M* = 422 geographical units of regional populations with a mean of 1663 individuals (s.d. = 387) each. We assumed that these census data still represent reasonably well the extant distribution of people across the study area for providing general insights into disease spread across the urban–rural gradient. However, the urban area of Swansea experienced an increase of 1000–2200 national and international immigrants per year since the last census (Swansea City Council website, accessed 22 October 2020), which probably resulted in a slight increase in the urban–rural gradient in population sizes.

We used a gravity model to define the connections between populations, as it is capable of reflecting the connectivity underpinning landscape-scale epidemics [11–13]. In particular, a gravity model was chosen as the LSOA administrative units are characterized by fairly similar population sizes, although they can have different population densities because of different spatial extents of the underlying areas. We calculated for each pair of populations a gravity measure *T_i,j_* of the relative strength of how individuals are attracted to population *i* from populations *j* by accounting for local population sizes *N* and weighted pairwise Euclidean distance measures *d^ζ^*, including the 10 nearest populations *k* of the attractive population

2.1 $$T_{i,j}=\;\frac{\log\;\left(N_{i}+\;\sum_{k=1}^{10}(N_{k}/d_{i,k}^{\zeta })\;\right)\times \text{log}\;(N_{j})}{d_{i,k}^{\zeta }}.$$

We assumed that this approach reflects reasonably well situations in which people are most attracted to higher density population clusters of urban populations (i.e. Swansea in our case study; the arbitrary selected number of 10 nearest populations generates larger values of *T_i,j_* if the attractant population is closely surrounded by others; electronic supplementary material, figure S1). The scaling factor *ζ* (0 ≤ *ζ* ≤ 1) is a sampled parameter that may vary across scenarios, accounting for the uncertainty in population connectivity. For each population *i*, we computed a regional gravity index (with self-terms of $T_{i,j}^{*}$ for *i* = *j* being zero),

2.2 $$c_{i}=\;\overset{M}{\underset{\;j=1}{\sum }}T_{i,j}^{*},$$

based on the scaled (mean subtracted from values divided by 1 s.d.) values of *T_i,j_* (denoted $T_{i,j}^{*}$), which we assumed to reflect the overall connectivity of the population within the global metapopulation. We used values of $T_{i,j}^{*}$ multiplied by the commuter travel frequency among populations (*ρ*) to compute the number of individuals visiting each population from elsewhere.

Within each local patch in the metapopulation, individuals encounter each other depending on their social interactions. The daily within-population contact numbers *F_i,t_* for any individual *i* at time *t* is assumed to be a random draw given by the sum of contacts drawn from a negative binomial (with *r* = 3 and *p* = 0.26, resulting in contact numbers with mean = 9 and s.d. = 6) and a lognormal distribution (with mean = 3 and s.d. = 2, resulting in additional contact numbers with mean = 12 and s.d. = 16), whereby the lognormal distribution accounts for the ‘long tail' of contact frequency distributions. These parameters were based on a previous study of social contact frequencies in the UK [14]. For simplicity, and having in mind the main focus of this study on metapopulation-level patterns of disease spread, we did not account for repeated contact with the same individuals such as household or group members over different days. For simplicity, commuting individuals were assumed to return to their home populations in each time step, and their contacts were drawn in the same way as for non-commuting individuals.

### 2.2. Modelling the outcome of different disease control strategies in variable disease landscapes

We ran numerical simulations of an individual-based stochastic difference equation S–E–A–I–R model at daily time steps (see electronic supplementary material), with individuals transitioning from a (S)usceptible compartment to being (E)xposed if infected. Exposed individuals become either infectious and symptomatic (I) or infectious but asymptomatic (A) after an incubation period of *τ* days. They then transition to a (R)emoved compartment with the recovery rate *γ*, which removes them from taking any further part in the transmission cycle. Both symptomatic and asymptomatic individuals can expose those susceptible to the virus.

The force of infection *λ_i,t_*, i.e. the probability that a susceptible individual *i* acquires SARS-CoV-2 at time *t*, is calculated by considering the probabilities of the virus being transmitted from any interacting infected individual *k* (with *k* ∈ 1, …, *K_i,t_,* and *K_i,t_* being the number of all infectious individuals in the randomly sampled daily contact number *F_i,t_* of individual *i*); *λ_i,t_* can be computed based on the probability that none of the contact events with an infectious individual leads to an infection

2.3 $$\lambda_{i,t}\;=1-\prod_{k\;\in \;\{1,\;\ldots ,\;K_{i,t}\}}\left(1-\beta_{\omega }k\right),$$

where *β* is the disease transmission parameter and *ω_k_* is a scaling factor of infectiousness of asymptomatic relative to infectious individuals with 0 < *λ_i,t_* < 1.

To explore different scenarios of local and global epidemic sizes, we accounted for different pandemic stages and uncertainty in epidemiological parameters by systematically varying the following six parameters (see electronic supplementary material, table S1):

(1) transmission parameter (*β*),
(2) the proportion of individuals that remain asymptomatic after infection (*φ*),
(3) the relative infectiousness of asymptomatic disease carriers (*ω*),
(4) commuter travel frequency of individuals between populations (*ρ*),
(5) density dependence of individual contact numbers (*δ*),
(6) proportion of the overall population resistant/recovered from infection at the onset of simulations.

Density dependence of contact numbers (a population-level attribute) was modelled by calculating the scaled regional population density (i.e. all values divided by maximum density) to the power of the parameter *δ* and multiplying the corresponding values by the lognormal (long-tail) component of the daily contact numbers *F_i,t_*. The resulting value corresponds to the same contact frequencies if *δ* approaches zero and truncated contact frequencies at low population densities if *δ* approaches 1. Owing to the lack of better empirical evidence, we assumed this approach to represent the situation in which an increase in population density (in urban areas) can result in a larger overall number of random encounters between citizens and higher contact frequencies between individuals of the same community in urban areas [15].

To assess and compare the efficacy of different, idealized, disease control strategies, we defined three general control strategies:

(i) Trace and isolation of any infected individuals with a certain proportion (*κ*) of all infected individuals successfully isolated (removal of individuals in disease states E, A, I, reflecting scenarios where intensive and continuous testing and/or intensive contact tracing would allow removal of any infected individuals; termed ‘trace all' in figures).
(ii) Trace and isolation of symptomatic individuals only with a certain proportion (*ɛ*) of symptomatic individuals successfully isolated (removal of individuals in disease state I, reflecting scenarios where symptomatic cases isolate without any additional contract tracing or testing; termed ‘trace symptomatic only' in figures).
(iii) Regional temporary reduction in transmission rates (regional lockdown) in response to a regional outbreak within the modelled LSOA administrative units, with four parameters to vary for decision-making and control: (1) a threshold *α* defining the proportion of the regional population to be in disease state I, (2) lockdown stringency *ϕ* (the factor by which the transmission parameter is reduced), (3) travel ban distance *ν* (the maximum distance from which individuals are allowed to visit a locked-down population), and (4) duration of regional lockdown (*η*).

For simplicity, we did not account for possible individual heterogeneity in transition probabilities between different disease states but rather assumed constant ‘average' transition probabilities in each scenario, albeit waiting times at different disease states are heterogeneous for many infectious diseases [16]. Similarly, we assume that the delay in the detection of individuals in different disease states is covered in the ‘average' parameter of tracing/removing these individuals from transmission cycles as part of control strategies.

It should also be noted that parameters concerning the efficacy of control strategies, such as the proportion of individuals traced and isolated or lockdown stringency, represent the realized rather than the system-inherent efficacy. It is expected that system-inherent efficacy of control measures, such as the technical capacity to trace infectious individuals, is highly dependent on compliance by the public. However, because of the lack of accurate information on compliance under different circumstances available, we did not account for compliance as an independent parameter in our simulations. A particular aim of our study was to investigate the interaction between this realized efficacy and the patterns of response across the urban–rural gradient. We are aware that real-world heterogeneity in the transition between disease states, or heterogeneity in contact patterns arising from unpredictable super-spreading events (e.g. large social gatherings), constitute unknown factors that might impose some of the intricate challenges of disease control. Without further evidence as to how to account for this in our model, however, we decided to keep it as simple as possible rather than introducing a large number of unpredictable events that can potentially modulate transmission dynamics. We do so as, here, we are solely interested in population-level outcomes of SARS-CoV-2 spread in response to different control strategies.

### 2.3. Numerical simulations

To be able to assess the efficacy of these control strategies when compared with a reference, we defined 10 000 ‘baseline' transmission scenarios by varying the epidemiological parameters defining the spread scenarios (1–6 above). We performed independent numerical simulations for each parameter combination. We then combined each baseline transmission scenario with varying parameters for each of the three control strategies, running a total of 40 000 simulations, each for a time period of 100 days, which we assumed to be sufficiently long to capture the epidemic dynamics in response to different parameter values. Parameter values were sampled using Latin hypercube sampling [17]; see electronic supplementary material, table S1 for the ranges of parameter values used. Our simulations are stochastic in that any individual's transition in epidemiological state or whether an individual is traced and removed are random Bernoulli draws based on the given parameter values.

We started each simulation by randomly allocating *n* = 422 individuals as infectious (corresponding to the number of populations, but not necessarily one infectious individual in each population and infectious individuals are not necessarily seeded in high-density populations) in the metapopulation. While this seeding of the epidemic does not represent any particular ‘true' epidemic state in the studied population, we have chosen this seeding together with the varying number of initially resistant proportion of populations to enable us to explore different scenarios of dynamic disease landscapes rather than any particular past or current state.

### 2.4. Output summary

For each simulation, we computed the epidemic sizes as the numbers of individuals that had been symptomatic (we considered symptomatic cases only as asymptomatic cases are less likely to result in hospitalization or any other severe health burden) for each population and at the metapopulation scale (i.e. entire population). In order to explore the sensitivity of different control strategies to different epidemiological parameters, we calculated the relative differences in epidemic sizes (relative epidemic size) for each disease control scenario and the corresponding baseline scenario at the regional and metapopulation scale such that values close to zero mean effective control and larger values mean less effective control. Moreover, we computed for each baseline scenario the strength of correlation (expressed as the *r* value from the Spearman rank correlation) between the regional relative epidemic size and the respective regional gravity index (urban–rural gradient in relative epidemic size) in order to explore whether control strategies varied in their efficacy across urban–rural gradients. A strong positive correlation can be interpreted as a strong urban–rural gradient of disease spread, with smaller relative epidemic sizes in rural areas, where connectivity is generally lower. We also computed the strength of correlation between the epidemic sizes of baseline scenarios (uncontrolled outbreaks) and the respective regional gravity index.

In order to explore variation in the relative epidemic size and the efficacy of different control strategies for different scenarios, we used generalized linear models (GLMs) and boosted regression trees (BRTs) as implemented in the R package *dismo* [18]. We express results in terms of the direction of effects (i.e. decrease/increase in relative epidemic size, reflecting higher/lower control efficacy) and relative influence (i.e. percentage of variance explained by various parameters in the corresponding BRT model) for those parameters that appear to show ‘significant' effects in both GLMs and BRTs (i.e. GLM coefficients clearly distinct from zero, relative parameter influence greater than 5%).

All analyses and plotting were conducted in R v. 4.0 [19].

## 3. Results

The urban–rural gradient in epidemic sizes (expressed as the rank correlation coefficient between the regional epidemic size and the regional gravity index) considerably decreased among baseline scenarios (uncontrolled outbreaks) with larger transmission parameters (*β*, explaining 57% of changes in total epidemic sizes). This indicates that larger outbreaks concentrated in urban areas occur mostly at low transmission parameters. In addition, the urban–rural gradient in total epidemic sizes decreased with higher commuter travel frequency ( *ρ*, 19% of changes in total epidemic sizes) and stronger distance weighting in the underlying gravity model (*ζ*, 15% of changes in total epidemic sizes). This suggests that these factors not only facilitate spatial disease spread but also determine whether outbreaks are larger in urban than in rural environments.

### 3.1. Efficacy of different control strategies in changing disease landscapes

**Figure 2.**
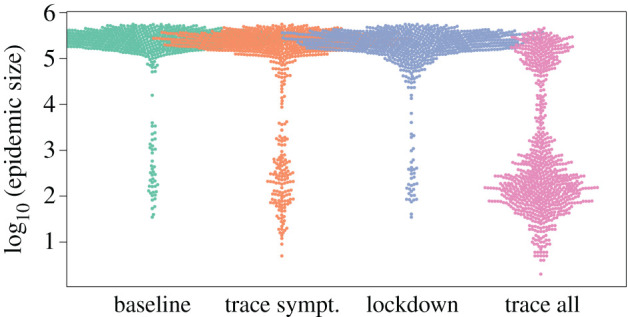
Distribution of the total COVID-19 epidemic sizes across an urban–rural gradient. The plot shows log_10_-scale epidemic size at the metapopulation level resulting from simulating a large range of scenarios. Scenarios include: ‘baseline': no control strategy; ‘trace sympt.': isolation of a certain percentage of infectious/symptomatic virus carriers only; ‘lockdown': regional reduction in transmission parameters in response to a certain number of infectious/symptomatic virus carriers being present; ‘trace all': isolation of a certain percentage of infected individuals (i.e. those in the disease states exposed, asymptomatic virus carriers or infectious/symptomatic virus carriers). To aid visualization, the plot is based on a random selection of 10 000 out of 40 000 simulation results.

**Figure 3.**
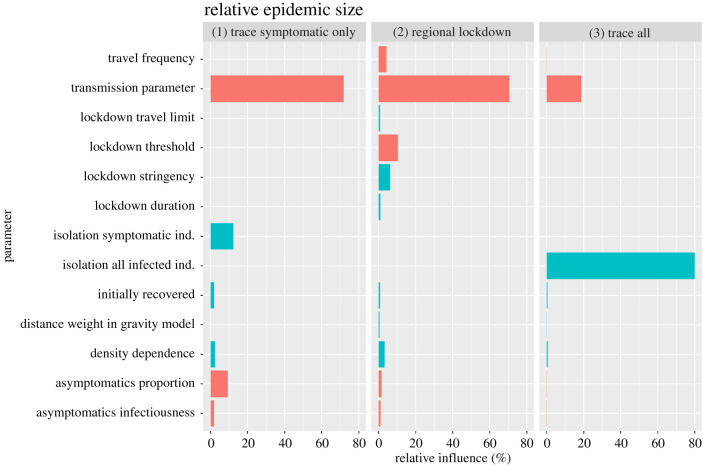
Relative influence of different parameters on the relative epidemic sizes*.* Relative epidemic sizes were calculated for simulations with three different control strategies compared with baseline scenarios of no COVID-19 control. The three different control strategies were ‘trace symptomatic only': isolation of a certain percentage of infectious/symptomatic virus carriers only; ‘regional lockdown': regional reduction in transmission rates in response to a certain number of infectious/symptomatic virus carriers being present; and ‘trace all': isolation of a certain percentage of individuals being infected in the disease states exposed, asymptomatic virus carriers or infectious/symptomatic virus carriers. Green bars indicate smaller and red bars larger relative epidemic sizes with increasing parameter values.

*Trace and isolation of all infected individuals* (trace all) was by far the most efficient control strategy in our simulations (figure 2): no simulated scenario with 47% or higher of infected individuals removed (*κ*) had a relative epidemic size greater than 5% of the respective baseline scenario. Lowering the epidemic size through isolation of infected individuals was less efficient for large transmission parameters (*β*, explaining 19% relative influence on changes in relative epidemic sizes, figure 3).

*Trace and isolation of symptomatic individuals* (trace symptomatic only) was of limited efficacy in lowering epidemic size in our simulations. The efficacy of these control strategies largely depends on small transmission parameters (*β*, 72% relative influence), whereas variation in the proportion of symptomatic individuals being isolated (*ɛ*) explained only 12% in relative epidemic sizes. The efficacy of this control strategy was further hampered by increasing proportions of asymptomatic cases (φ, 9% relative influence).

*Regional lockdown scenarios* appeared to be of limited efficacy in our simulations (figure 2) and largely depend on small transmission parameters (*β*, 70% relative influence) (figure 3). Their efficacy was sensitive to the regional threshold levels for lockdown implementation (*α*, 10% relative influence) and lockdown stringency (*ϕ*, 6% relative influence). A reduction in relative epidemic sizes to 5% of those of the respective baseline scenarios through regional lockdowns was only achieved for regional lockdown threshold levels of less than or equal to 1% of the populations being symptomatic.

### 3.2. Variation in control efficacy across urban–rural gradients

**Figure 4.**
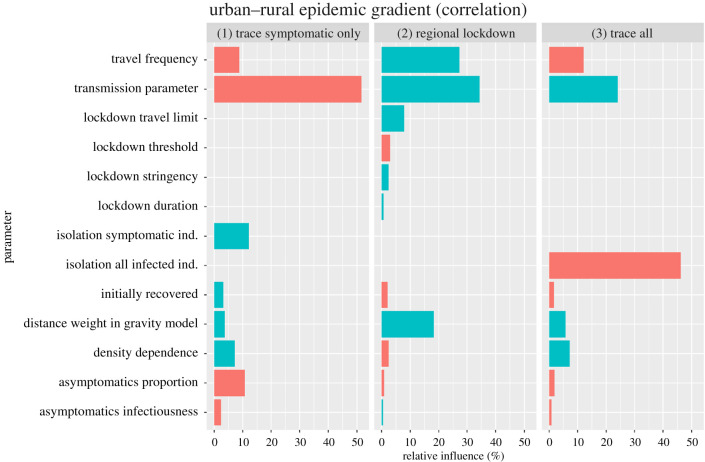
Relative influence of different parameters on the ‘urban–rural gradient' (correlation coefficients of regional relative epidemic sizes with connectivity across all populations). Stronger correlations mean larger regional epidemic sizes in populations with increased connectivity, which are typically urban areas. Relative epidemic sizes were calculated for simulations with three different control strategies compared with baseline scenarios of no COVID-19 control. The three different control strategies were ‘trace symptomatic only': isolation of a certain percentage of infectious/symptomatic virus carriers only; ‘regional lockdown': regional reduction in transmission rates in response to a certain number of infectious/symptomatic virus carriers being present; and ‘trace all': isolation of a certain percentage of individuals being infected in the disease states exposed, asymptomatic virus carriers or infectious/symptomatic virus carriers. Green bars indicate decreases and red bars increases in correlation strength with increasing parameter values.

**Figure 5.**
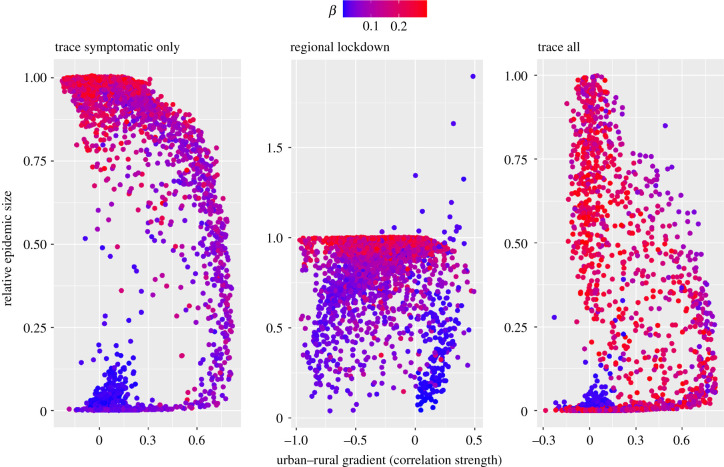
Relationship between overall relative epidemic size for different control measures and the underlying urban–rural gradient in epidemic size arising from different simulation scenarios. Relative epidemic sizes were calculated for each simulation with three different control strategies compared with baseline scenarios of no COVID-19 control. The urban–rural gradient in epidemic sizes was computed as the strength of correlation with the regional relative epidemic size and the respective population-level connectivity index. The three different control strategies were ‘trace symptomatic only’: isolation of a certain percentage of infectious/symptomatic virus carriers only; ‘lockdown’: regional reduction in transmission rates in response to a certain number of infectious/symptomatic virus carriers being present; ‘trace all': isolation of a certain percentage of individuals being infected in the disease states exposed, asymptomatic virus carriers or infectious/symptomatic virus carriers. Each point represents the outcome from a simulation with a different baseline scenario (i.e. combination of different input parameter values), coloured according to the respective value of transmission parameter (*β*).

The strength of the urban–rural gradient in relative epidemic sizes resulting from *isolation of all infected individuals (E, A, I)* declined with increasing proportions of infected individuals isolated (*κ*, 46% relative influence; figure 4) and increased with increasing transmission parameters (*β*, 24% relative influence), suggesting that larger transmission rates makes it relatively more challenging to control the spread in urban than in rural areas. By contrast, the more individuals are isolated (increasing *κ*), the more efficiently that epidemics can also be contained in urban environments (i.e. resulting in less strong urban–rural gradients in relative epidemic size), despite a concentration of cases there, as depicted by mostly positive correlation coefficients in the urban–rural gradient in relative epidemic size (figure 5).

The completely opposite effect was found for the *isolation of symptomatic individuals only* (*I*). The strength of the urban–rural gradient in relative epidemic size declined with increasing transmission parameters (*β*, 52% relative influence) but increased with increasing proportions of symptomatic individuals isolated (*ɛ*, 12% relative influence). Hence, larger transmission rates make a reduction in epidemic size by isolation of symptomatic individuals only more challenging in rural rather than in urban areas. The urban–rural gradient in relative epidemic size further decreased with larger proportions of asymptomatic cases (φ, 11% relative influence), decreased with higher commuter travel frequency ( *ρ*, 8% relative influence) and increased with stronger density dependence in contact numbers (*δ*, 7% relative influence, figure 4).

In response to *regional lockdown* strategies, the strength of the urban–rural gradient in relative epidemic size increased with increasing transmission parameters (*β*, 34% relative influence), increasing travel frequencies (27% relative influence) and stronger distance weighting in the underlying gravity model (*ζ*, 18% relative influence, figure 4).

## 4. Discussion

Decision-making to balance efficient COVID-19 control with socio-economic pressures is a challenging task against the backdrop of asymptomatic disease spread and ever-changing disease landscapes. We show that isolation of symptomatic cases or regional lockdowns in response to local outbreaks have limited efficacy in terms of reducing overall epidemic sizes, unless the overall transmission rate is kept persistently low. Isolation of non-symptomatic infected individuals, who may be detected by effective test-and-trace approaches, is pivotal to reducing overall epidemic size over a wider range of transmission scenarios. By considering an ‘urban–rural epidemic gradient' as the strength of correlation between regional epidemic size and connectivity within a region, we show that, under certain conditions, control measures are of limited efficacy in urban compared with rural areas. Intervention strategies focusing on the isolation of non-symptomatic individuals and regional lockdowns, for example, had the strongest urban–rural outbreak gradients at high transmission rates. By contrast, interventions targeting symptomatic virus carriers only had the reverse effect.

Our results emphasize the importance of efficient detection of infectious individuals through test-and-trace approaches for containing the spread of SARS-CoV-2 [2,20,21], while also uncovering that some methods will be less efficient in urban areas under the post-lockdown situation unless transmission rates are kept constantly low.

Efficient removal of all infectious individuals (including non-symptomatics) has the potential to restrain total epidemic size by successfully suppressing landscape-scale disease spread and the corresponding source–sink dynamics of how the disease may spread and re-emerge among populations. We found regional lockdowns to only be effective in terms of reducing overall epidemic size if implemented at low threshold levels and low transmission rates. This is likely to be due to the fact that only under these conditions can landscape-scale spread of the disease be avoided. These findings are in line with previous suggestions that temporary lockdowns do not necessarily contain overall epidemic size in a metapopulation context over medium to long time periods [22], even if they may be useful for reducing local case number over short time periods to avoid an overload of health capacities [23–25].

In practice, the prominent example of the locally restricted lockdown implemented in the city of Leicester in the UK, which began in June 2020, is just one example of mounting evidence that regional lockdowns do not necessarily see a reduction in disease transmission during the following weeks [26], which would ideally prevent spread of the virus beyond the local context. This slow response of incidence decline following regional lockdowns is in line with our finding and more general suggestions that disease with asymptomatic transmission pathways can only be controlled with intensive test-and-trace approaches [27]. A number of natural experiments are being conducted during the course of the epidemic in the UK (at the time of writing in October 2020) on locally tiered measures, with a range of prevalence in the background, which will provide data to evaluate this intervention going forward.

Surprisingly, we found travel frequency and possible density dependence in contact frequency to have rather small relative impact on overall epidemic size compared with the transmission parameter (figure 3). Despite the recognized importance of connectivity, travel patterns and metapopulation structure on disease spread [28–30] our results highlight the importance of overall transmission rates on disease spread and epidemic size. This has important management implications, as it points to measures that might allow for continuous long-term lowering of transmission rates. Such measures, we suggest, are considerably more efficient than any short-term measures of changing control stringency in response to actual case numbers for reducing the overall epidemic size.

We found the magnitude of the transmission rate to also determine the success of different control strategies in urban versus rural areas, leading to varying urban–rural epidemic gradients in response to varying transmission rates and different control strategies (figure 4). For interventions focused on isolating both non-symptomatic and symptomatic individuals and regional lockdowns, our results reveal the strongest urban–rural epidemic gradients at high transmission rates, indicating a reduced efficacy of such control measures in urban areas under these conditions. These results suggest that, at high transmission rates, the urban–rural epidemic gradient is enforced by the overall poorly curbed disease spread at the metapopulation level (figure 5). Conversely, we found the urban–rural gradient in epidemic sizes to be mostly masked at high transmission rates for measures targeted at symptomatics only, suggesting that these measures (which are generally of moderate to low efficacy) would not contain disease spread at the metapopulation level unless transmission rates are kept constantly low (figure 4). Exploring such effects warrants further investigation based on empirical data and relevant spatio-temporal models of disease spread under variable conditions of contact frequencies and control efforts. Such more detailed research may also account for first insights into variable compliance in response to intervention strategies. A recent study, for example, found slightly larger reductions in average mobility in high-density than in low-density areas in the UK [31].

In contrast to many forensic COVID-19 models that have focused on forecasting real outbreaks in specific locations [1,9,10] our model is strategic, with a focus on exploring general mechanisms emerging from across a large range of modelled scenarios. A direct match to the ongoing epidemic in the study area is unfeasible because we do not account for any particular real-world starting conditions nor the temporary changes in human interactions in response to changing policy. Also, as we are not aware of detailed estimates of relevant epidemiological parameters such as how transmission rate varies among age groups in our study area, we do not account for age structure in our model, even though, as it has been shown, COVID-19 effects and expression of symptoms are rather different between children and adults [32]. These effects might be exacerbated by a potential systematic variation in demographic community composition in urban and rural areas. However, with an area-wide spread of COVID-19 in our study area and a concentration of cases in urban communities during the first six months of the epidemic, some general patterns found in model output and empirical data appear to be compatible (K. Wells 2020, personal observations). Given more detailed data of spatio-temporal disease spread and better estimates of epidemiological key parameters, future studies may narrow down the currently intractable large parameter space through statistical approximation methods in order to identify when and how management efforts may results in disease extirpation versus long-term persistence [33]. Future studies may also account for the various processes that synergistically determine control efficacy and whether a certain level of control can be achieved or not. While our strategic modelling approach accounts only for ‘average' parameter values, possible sources of variation in the efficacy of control may include forward versus backward test-and-trace efficacy (i.e. tracing known social contacts versus previous contact from which the infection has originated [34]), disproportionate disease spread by super-spreading individuals and super-spreading events, and dynamic changes in compliance by the public.

The most important implication from our model is that priority should be given to any reliable and feasible measures that constantly keep the transmission rate low as opposed to relying on local lockdowns to stamp out outbreaks. The success of any short-period interventions is limited if overall transmission rates remain high and facilitate disease spread within and among populations. We conclude that, in the absence of an intervention strategy that would ensure rapid eradication of COVID-19, different intervention strategies do not work as efficiently in urban as in rural communities. Priority should thus be given to further research on how the most vulnerable individuals can be best protected at minimal cost for entire metapopulations. While post-lockdown situations of low transmission rates and reduced case numbers are tempting to ease interventions, we believe that ongoing source–sink dynamics of disease spread cannot be ignored. Control strategies aiming at successful regional disease control during a pandemic should not ignore the fact that communities that successfully escaped initial epidemic waves remain highly vulnerable because they contain large pools of individuals still susceptible to COVID-19.

## Supplementary Material

Supporting information

## Data accessibility

The R code and data for this study can be found on GitHub https://github.com/konswells1/COVID19-LSOA-metapopulation-model.
