# Supplementary material for: Disease control across urban–rural gradients
Source: J R Soc Interface. 2020 Dec 9;17(173):20200775. doi: 10.1098/rsif.2020.0775 (PMC7811581; doi:10.1098/rsif.2020.0775)
Supplement: Supporting information [file rsif20200775supp1.docx]

**Supporting information**

**Disease control across urban-rural gradients**

Authors: Konstans Wells, Miguel Lurgi, Brendan Collins, Biagio Lucini, Rowland R. Kao, Alun L. Lloyd, Simon D.W. Frost, Mike B. Gravenor

Journal: *Journal of The Royal Society Interface*

**Model framework**

We used a stochastic individual-based simulation model of disease spread in a metapopulation of connected local populations over a time period of 100 daily time steps.

The purpose of this model is to simulate the spread of COVID-19 in a metapopulation that resembles an urban-rural gradient in terms of population structure as found in the southwestern part of Wales. We aimed to compare the outcomes of different disease control strategies in changing disease landscapes as given by the metapopulation context, the initial proportion of resistant individuals, and the fraction of infected individuals that do not develop symptoms after infection. We aimed to explore general patterns of disease spread in terms of epidemic sizes (total number of individual to become infected over the course of the simulated epidemics) and the variation of epidemic sizes in different urban and rural regions in response to different control strategies. We parameterise the epidemiological parameters of our model with currently available information about COVID-19 (first estimates of the time periods of incubation and being infectious). The R code for this study can be found on GitHub <https://github.com/konswells1/COVID19-LSOA-metapopulation-model>.


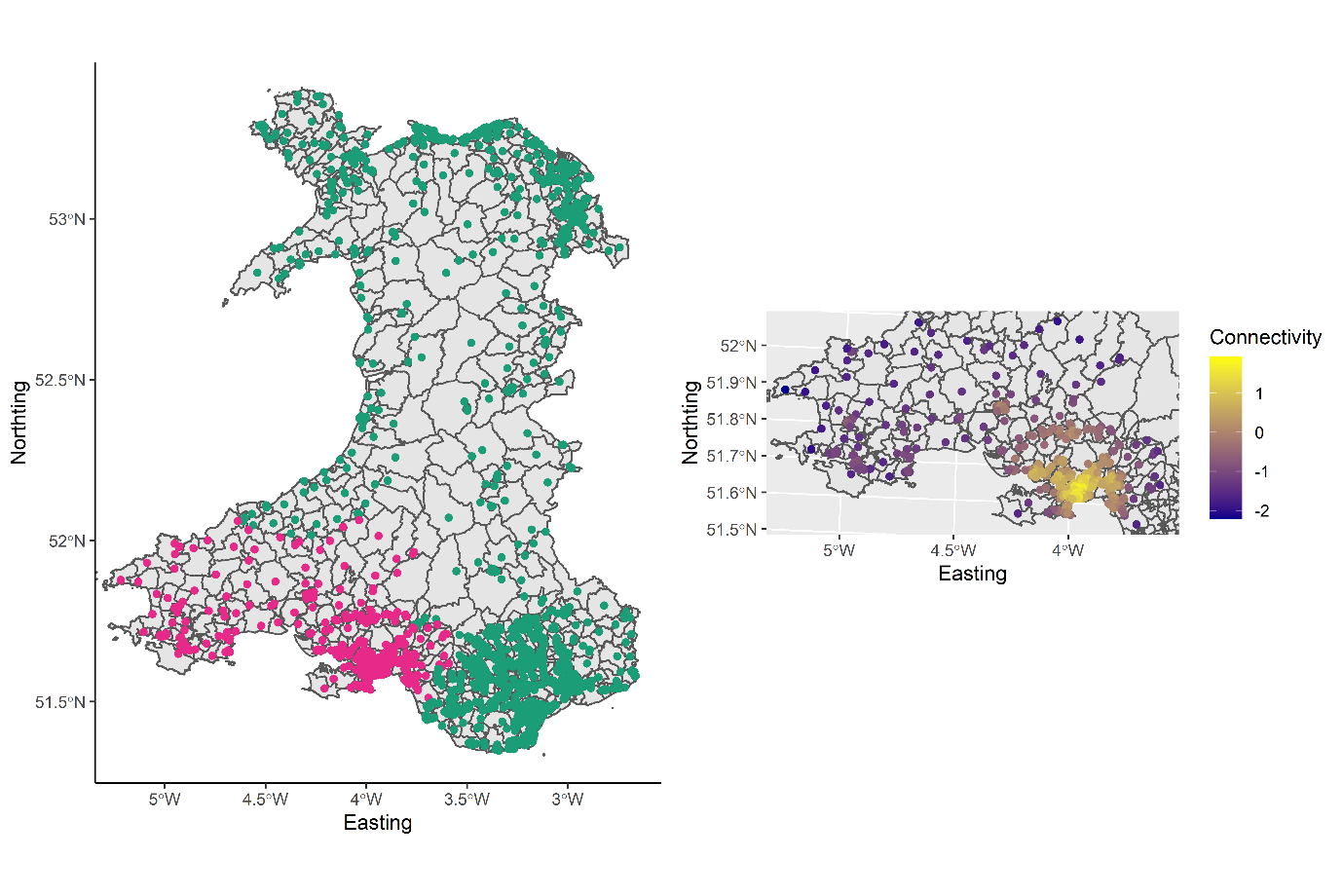


**Figure S1**. **Maps showing the study area**. Red points in the left panel/map indicate the selected study area of four counties in southwestern Wales, the right panel/map shows the selected 422 LSOA populations coloured according to the gravity index values (yellow: urban area with high connectivity, blue: rural area with low connectivity).

**Table S1**. Parameter definitions and their values / sampled ranges for the individual-based model of Covid-19 disease spread in a metapopulations resembling southwestern Wales. Parameters sampled with changing values (drawn from uniform distributions) in each scenario are marked with an asterix. The parameter ranges are largely defined based on values retrieved from the literature or, if unknown, from sufficiently large possible parameter ranges.

| **Parameter** | **Symbol** | **Range/ unit** | **Description** |
| --- | --- | --- | --- |
| Metapopulation size | *N* | 701,995 | Total population size |
| Population number | *M* | 422 | Number of populations (LSOA administrative units) within the metapopulation. |
| Transmission rate | *β* | 0.001 – 0.3 | Transmission rate of the virus/disease of a susceptible individual interacts with an infectious individual |
| Incubation period | *τ* | 4 – 6 days | Time between infection of an individual and the onset of being infectious [31]. |
| Recovery rate | *γ* | 1/10 – 1/7 | Daily recovery rate (inverse of the timespan until recovery of infectious individuals). |
| Proportion of individuals that remain asymptotic after infection | *ϕ* | 0.1 – 0.7 | Proportion of individuals that do not develop symptoms after infection but may be infectious. |
| Infectiousness of asymptomatic disease carriers | *ω* | 0.5 – 1 | Scaling factor of infectiousness of asymptomatic individuals compared to infectious individuals. |
| Proportion of individuals resistant/recovered at onset of simulation | *μ* | 0.1 – 0.5 | Proportion of individuals resistant/recovered at onset of simulation, i.e. in disease state ‘R’. |
| Density-dependence in contact frequencies | *δ* | 0 - 1 |  |
| Commuter travel frequency among populations | *ρ* | 0.001 – 0.2 | Travel rate (commuter travel) to connected populations (direct connection with a link between two populations) in a graphical network. |
| Scaling factor of population connectivity | *ζ* | 0 - 1 | Scaling factor (exponential) of distance in the gravity model that quantifies the connectivity of populations in each simulation. |
| ***Parameters of control strategies*** | | | |
| Proportion of all infected individuals (E,A,I) isolated | *κ* | 0 – 0.9 | Proportion of all infected individuals isolated each day in scenarios with tracing of all infectious individuals (disease states ‘E’, ‘A’, or ‘I’). This may include all individuals that transitioned any time before the previous day into relevant disease states. |
| Proportion of all symptomatic individuals (I) isolated | *ε* | 0 – 0.9 | Proportion of all symptomatic individuals isolated each day in scenarios with tracing symptomatic individuals only (disease state ‘I’). This may include all individuals that transitioned any time before the previous day into into this state. |
| Threshold level for regional ‘lockdown’ | *α* | 0.01 – 1.0 | Threshold level of % population being symptomatic before regional lockdowns are implemented. |
| Lockdown stringency | *φ* | 0.5 - 1 | Scaling factor for lowering β during regional ‘lockdown’, here set to constant value. |
| Time period of regional lockdown | *η* | 10 – 30 days | Length of time period of mitigation efforts; all mitigation efforts are assumed to start 7 days after the introduction of symptomatic/infectious individuals and onset of simulations. |
| Maximum travel distance during regional ‘lockdown’ | *ν* | 5 – 20 km | The maximum distance from which individuals are allowed to visit a locked-down population. |
